# Relationship between functional independence and community integration of people with spinal cord injury in Bangladesh

**DOI:** 10.3389/fresc.2024.1435656

**Published:** 2024-12-11

**Authors:** Shazal Kumar Das, Md Bakhtiar, Saiba Muhammad Sabrin, Michael Curtin, Ehsanur Rahman, Zahid Bin Sultan Nahid, Zakia Rahman, Md. Furatul Haque, Md. Fazlul Karim Patwary, Md. Jahangir Alam, Md. Emran Hossain, Md. Atiar Rahman, Shafiqul Islam, Md. Ashfaquzzaman, Md. Anowar Khasru Parvez

**Affiliations:** ^1^ Department of Physiotherapy, Bangladesh Health Professions Institute, Dhaka, Bangladesh; ^2^ Department of Physiotherapy, Khwaja Badrudduja Modern Hospital, Gazipur, Bangladesh; ^3^ School of Allied Health, Exercise and Sports Sciences, Charles Sturt University, Albury, NSW, Australia; ^4^ Department of Physiotherapy, Jashore University of Science and Technology, Jashore, Bangladesh; ^5^ Department of Physiotherapy, SAIC College of Medical Science and Technology, Dhaka, Bangladesh; ^6^ Department of Microbiology, Jahangirnagar University, Dhaka, Bangladesh; ^7^ Department of Physiotherapy, Mymensingh College of Phsyiotherapy and Health Science, Mymensigh, Bangladesh; ^8^ Department of Physiotherapy, Centre for the Rehabilitation of the Paralysed, Dhaka, Bangladesh; ^9^ Department of Physiotherapy, Chittagong Medical College, Chittagong, Bangladesh

**Keywords:** prospective, community integration, spinal cord injury, cross sectional study, functional independence

## Abstract

**Design:**

Prospective, cross-sectional study.

**Objectives:**

To determine the functional outcome and home and social integration of people who had spinal cord injury and completed their inpatient rehabilitation.

**Setting:**

Centre for the Rehabilitation of the Paralysed (CRP), Bangladesh.

**Methods:**

Spinal Cord Independence Measure (SCIM) and Community Integration Questionnaire (CIQ) were used to analyse the relationship between the functional outcome and home and social integration at the end of rehabilitation. Descriptive and inferential statistics were performed to analyse the data.

**Results:**

A total of two hundred participants (181 men and 19 women) were recruited for the study. Among the participants, 92.5% of them reported a history of trauma or accident, including road traffic accidents, falls and other injuries. Approximately 60% of participants presented with paraplegia and 62.5% of participants were categorized on the ASIA Impairment Scale (AIS) as Grade A, complete spinal cord injury. Participants with paraplegia and participants with a Grade B, incomplete injury, on the AIS were functionally more independent (*p* = 0.011)) compared with participants with tetraplegia and other AIS grades. Participants with paraplegia reported having a more active lifestyle (*p* = 0.040) in their home and social activities compared to those with tetraplegia. There was no significant association found between functional independence at pre-discharged and community integration one-month post-discharge of the people with SCI.

**Conclusion:**

A month after discharge, there is no statistically significant relationship between community reintegration and functional independence. A measure of functional independence may not be a suitable indicator of community integration. It is proposed that to monitor a person's community integration the CIQ could be used with a measure of quality of life as this would indicate a person's contentment with their level of community integration.

## Introduction

A spinal cord injury (SCI) can result in significant levels of impairment and lifestyle disruption (1, 2). The functional independence of people with a SCI is significantly lower than that of the population in general as a SCI usually causes severe locomotor impairment, due to paralysis of the muscles. Depending on the level and completeness of the lesion, a person with SCI can be independent or require assistance with a range of everyday activities (3). Secondary conditions and complications, such as age, concomitant injuries, level of injury, injury mechanism, pressure ulcers, urinary and/or respiratory infections, pain, and severity of spasticity, may impact on the level of independence of a person with a SCI (4) and in turn affect the outcome of their community re-integration (5, 6).

Loni et al. (7) have suggested that because of improved medical and rehabilitation services, more people with a SCI are surviving and their life expectancy has increased. In-patient rehabilitation following a SCI focuses on assisting a person to obtain a level of functional independence (7). An effective rehabilitation program can reduce patient-associated costs (e.g., length of hospital stay), lead to some level of independence (8, 9), and enhance overall quality of life (7).

A key marker of successful rehabilitation following spinal cord injury/disease is to facilitate the transition from inpatient hospital care to being able to participate in a range of roles back in their community (10, 11). Community re-integration refers to a person with SCI being able to participate in community life and being able to effectively perform their roles in community settings (11, 12). Hitzig et al. (10, p. S80) suggests that “meaningful participation in occupations or employment and/or the ability to engage in societal roles holds significant implications for one's health and wellbeing”. Many people with SCI find it difficult to transition from the supportive and accessible environment of the hospital and inpatient rehabilitation setting back into their community (10, 13, 14).

It is suggested that to enable successful community re-integration inpatient programs need to develop the self-efficacy and ability to manage their own life of person who has had a SCI (11). Successful community re-integration requires the translation of all the skills learnt as an inpatient into the community, the acceptance of family and friends of the person's decisions and choices, support from peers, having an accessible house, be able to move around the community, participate in work, leisure, and education activities of choice, and engage in satisfying social relationships (11, 13–16). Barclay et al. (17) found that successful community integration needs to take into consideration person factors (e.g., complications from the SCI such as fatigue, incontinence), physical and institutional environmental factors (e.g., transport issues, accessibility, inadequate support services), and social and cultural environmental factors (e.g., social networks, negative social attitudes, reliance on family and friends). Furthermore, social and community participation in which the person with SCI is establishing and maintaining close relationships is key to successful community integration (13).

As the incidence of SCI is significantly higher in non-developed countries compared with developed countries (18) it is important to consider the rehabilitation outcomes for people with a SCI who live in developing countries, where there may be limited services and resources for rehabilitation and community integration. Where rehabilitation is provided for people with a SCI who live in a developing country the outcome of successful community re-integration is expected as this is a common outcome of all SCI rehabilitation programs (10, 11). In Bangladesh, the Centre for the Rehabilitation of the Paralysed (CRP), is a specialized SCI facility that admits nearly 350 people each year (19). Rahman et al. (20) found that by the end of their in-patient rehabilitation at CRP many people with SCI had a positive perception of their level of function and their QOL. However, other authors found that post-discharge people with SCI experience an increase in functional limitations and a decrease in their QOL (21–23). Hossain et al. (21) concluded that most people with SCI who were living in the community, three to six years post-discharged from CRP were housebound, jobless, living in poverty, and had pressure ulcers. As a result of these findings, it is important to investigate the community re-integration of people with SCI who have completed their rehabilitation at CRP to inform and guide better outcomes.

At CRP there has been no research to date conducted to determine the relationship between functional independence at discharge and outcome of community integration. As the level of function independence at discharge has been shown to correlate with community integration (11, 13, 14, 16), it is proposed that, knowing the functional independence of people at discharge at CRP may provide some indication of the support they will require to re-integrate into their community providing a baseline for monitoring their support needs post-discharge. The focus of this study was to establish a baseline correlation between the functional independence of a person with a SCI at the end of their inpatient rehabilitation at CRP and their community re-integration one-month post-discharge.

### Research question

What is the relationship between functional independence at discharge and community integration one-month post-discharge of people with a SCI who attended in-patient rehabilitation at CRP?

## Methods

### Study design

A prospective cross-sectional study design was used to explore the functional outcome level of people who had SCI on completion of inpatient rehabilitation at CRP and their level of community integration (home and social) one-month post-discharge. This study was approved by the ethics committee of Centre for the Rehabilitation of the Paralysed, Bangladesh (CRP-R&E-0401-0401), and the Pabna University of Science and Technology, Pabna, Bangladesh (ERC/FBST/PUST/2022-116).

### Sample size

Men and women, 18 years of age or older, who has a spinal cord injury, were at the end of their inpatient rehabilitation at CRP, and who lived in a community relatively close to CRP, were invited to participate in the study. Due to funding issues, the study was only opened to participants who lived in a community relatively close to the CRP so that they could be easily followed up in person one month post-discharge. The recruitment process of study participants spanned approximately nine months and continued until 200 people who met the inclusion criteria agreed to participate.

### Measurement tools

The following demographic information was collected for each participant: age, gender, education level, marital status, employment, and type of injury. In addition, two survey instruments were used to collect data: the Spinal Cord Independence Measure (SCIM) and the Community Integration Questionnaire (CIQ).

The SCIM is a widely used instrument to measure functioning in everyday life activities of people with SCI (24). Fekete et al. (24, p. 40) stated that the main advantages of the SCIM over other functional assessments “are its sensitivity to changes in performance of tasks that are relevant for SCI patients, and the fact that it measures not only the burden of care, but also achievements, which have medical, psychological or social relevance for SCI patients”. This scale consists of 19 daily activity items arranged into three domains: mobility (9 items, scores ranging from 0 to 40), respiration and sphincter management (4 items, scores ranging from 0 to 40), and self-care (6 items, scores range from 0 to 20). A person's overall SCIM score can vary from 0 to 100, with higher scores representing better levels of competence or independence (20). The SCIM has been proven to be valid, responsive, and has excellent intra-rater and inter-rater reliability among rehabilitation professionals (25–27). Itzkovich et al. (28, p. 1926) stated that the SCIM was “an efficient measure for functional assessment of SCI patients and can be safely used for clinical and research trials”.

The CIQ provides a reliable and objective assessment of home and social integration and productive activities (29). The CIQ contains 15 items that assess community integration across three domains: home integration (scores range from 0 to 10 with questions relating to activities such as meal preparation, housework, and childcare); social integration (scores range from 1 to 12 points with questions relating to activities such as shopping, visiting friends, leisure activities); and productive activity (scores range from 0 to 7 points with questions relating to activities such as work, education, and volunteer activities) (30). A person's overall CIQ score can vary from 0 to 29 points. Gontkovsky et al. (31) found that the CIQ was a valid measure for examining community integration for people with SCI. In a systematic review conducted by Turcotte et al. (32) the CIQ was noted to have internal consistency for adults with SCI. Furthermore, Callaway et al. (29, p. 228) stated that the CIQ is frequently used in SCI research because it demonstrated “good criterion and construct validity, test-retest reliability, inter-rater reliability, and full-scale internal reliability”. For the purposes of this study only the domains of home integration and social interaction were used; hence the overall score varies from 0 to 22 points. It was decided not to include the productive activity domain because it did not match the study objectives. Higher scores indicate better community integration.

### Data collection procedure

Participants were recruited to the study towards the end of their in-patient rehabilitation. The SCIM was completed just prior to discharge and the CIQ was completed one-month later. The delay in completing the CIQ was to allow each participant a month to transition from in-patient rehabilitation back into their community. The SCIM took approximately 15–20 min to complete and the CIQ took approximately 10–15 min to complete. A member of the research team asked the questions and recorded each person's responses to assist with the accurate completion of the surveys.

### Data analysis

The Statistical Package for Social Sciences (SPSS), Windows version 22, (IBM, Armonk, NY, USA), was used to organize and analyse the data. Descriptive analysis, using frequency and percentage, was completed for different sociodemographic data: age, gender, education level, marital status, employment, and type of injury.

The Kolmogrov-Smirnov test was used to determine whether the SCIM and the CIQ data were normally distributed. As the data for both dependent variables did not have a normal distribution, the non-parametric inferential Mann-Whitney *U* and the Kruskal Wallis tests were used to analyse the data. The Mann-Whitney-*U*-test was used to test the homogeneity between two independent categories (i.e., age, gender, cause of injury and type of injury) (33) and the Kruskal–Wallis test was used to test the homogeneity between more than two independent categories (i.e., marital status, education, skeletal level of injury, ASIA scale, occupation and monthly income) (34).

Multiple logistic regressions were used to observe the impact of predictor variables (like socio-demographic and clinical characteristics) upon dependent variables (functional independence and community integration). Spearman correlation coefficient was performed to find the association between functional independence prior to discharge and community integration one month after discharge.

## Results

### Participant demographics and clinical information

Two-hundred people with SCI injuries participated in this study. The demographic and clinical characteristics of the participants are summarised in Table 1. The mean age of the participants was 35.68 ± 12.58 years. There were 181 men and 19 women respondents. Most of the participants identified as Muslim (*n* = 186), eleven identified as Hindu, and most of the participants were married (*n* = 152). Sixty participants completed primary education, 51 completed secondary school education, 23 completed higher secondary education, 25 completed a bachelor or higher degree, and the remaining 41 indicated they had not attended school. Thirty-five participants reported their occupation as farmer, 34 as day labourer, 36 as businessman, 15 as housewife, and 56 indicated they had other employment/occupations such as student, construction worker and rickshaw puller. Most of the participants had a monthly income between 100 and 200 United States Dollar (USD). Most participants had a traumatic SCI (*n* = 185), and 120 had a paraplegic injury. According to the American Spinal Injury Association (ASIA) Impairment Scale (AIS), 125 participants had complete A, 19 had incomplete B, 27 had incomplete C, and 29 had incomplete D spinal cord injury.

**Table 1:** Demographic and clinical characteristics of the participants.

Demographic% (*n*)		Demographic% (*n*)		Clinical characteristics% (*n*)	
Age		Education		Cause of injury	
<35 years	49 (98)	Never attended school	20.5 (41)	Traumatic	92.5 (185)
35 years & above	51 (102)	Primary Education	30 (60)	Non-traumatic	7.5 (15)
NB: Median (IQR) 35 years (25–45)		Secondary Education	25.5 (51)		
		Higher Secondary	11.5 (23)		
		Bachelor or above	12.5 (25)		
Gender		Occupation		Type of injury	
Male	90.5 (181)	Service holder	12 (24)	Paraplegic	60 (120)
Female	9.5 (19)	Businessman	18 (36)	Tetraplegic	40 (80)
Religion		Housewife	7.5 (15)	Skeletal level	
Muslim	93 (186)	Farmer	17.5 (35)	Cervical	34.5 (69)
Hindu	5.5 (11)	Day labourer	17 (34)	Thoracic	47 (94)
Buddhist	1.5 (3)	Others	28 (56)	Lumbar	18.5 (37)
Marital status		Monthly income (USD)		AIS scale	
Married	76 (152)	<100	24.5 (49)	Complete A	125 (62.5)
Single	20.5 (41)	100–200	68.5 (137)	Incomplete B	19 (9.5)
Divorced	3.5 (7)	>200	7 (14)	Incomplete C	27 (13.5)
		NB: Median (IQR) 100 (100–150)		Incomplete D	29 (14.5)

Abbreviations: *n*, number; AIS, American Spinal Injury Association Impairment Scale.

The ASIA Impairment Scale (AIS) is a means of classifying the severity of SCI (35). The AIS defines a complete SCI injury as “no preservation of motor and/or sensory function more than three segments below the neurological level of injury” and an incomplete SCI injury as “some preservation of sensory and/or motor function more than three segments below the neurological level of injury” (36, p. 3–4). The AIS uses the letters A–E to distinguish the different types of neurological injuries (Table 2).

**Table 2:** American spinal injury association impairment scale (AIS) (35, p. 1557).

Grade	Type of injury	Definition
A	Complete	No sensory of motor function in the S4–S5 sacral segments
B	Sensory incomplete	Sensory but not motor function below the neurologic level, including S4–S5 sacral segments
C	Motor incomplete	Motor function at most caudal sacral segments on voluntary anal contraction OR meets criteria for sensory incomplete and some sparing of motor function more than three levels below ipsilateral motor level on either side of body AND more than half of key muscles below neurologic level have a muscle grade less than 3.
D	Motor incomplete	Motor function below neurologic level AND at least half key muscles below neurologic level have a muscle grade of 3 or more.
E		Complete motor and sensory return; may have abnormal reflexes

### Spinal cord independence measure (SCIM)

The median score and *p*-value for each domain and the total score of SCIM associated with each demographic characteristic are presented in Table 3. Participants who never attended school and completed secondary and bachelor-level education had significantly higher scores with their functional independence level in the domains of self-care (*p* = 0.023), mobility (*p* = 0.013), and overall independence level. Participants with a paraplegic injury (lumbar skeletal level) had significant functional independence on mobility (*p* = 0.011) and overall independence (*p* = 0.042) measurement.

**Table 3:** Median scores and *P*-value of demographic and clinical factors for spinal cord independence measure (SCIM) domains.

Socio-demographic factors	Self-care		Respiration and sphincter management		Mobility (room and toilet) in bed and action to prevent pressure sores		Total SCIM score		
	Median	*P*-value	Median	*P*-value	Median	*P*-value	Median		*P*-value
Age^a^									
<35 years	4	0.631	11	0.92	4	0.223	23		0.141
35 years & above	4		10		3		22		
Gender^a^									
Male	4	0.452	11	0.17	4	0.871	22		0.972
Female	2		10		4		22		
Education^b^									
Never attended school	4	**0.023** *	10	0.84	5	**0.013** *	24	0.062	
Primary Education	3		10		3		22		
Secondary Education	4		11		3		23		
Higher Secondary	3		11		2		21		
Bachelor or above	4		10		8		24		
Type of injury^a^									
Paraplegic	4	0.791	11	0.75	4	**0.011** *	20	**0.042** *	
Tetraplegic	4		10		3		18		
Skeletal level^b^									
Cervical	4	0.321	10	0.510	3	**0.032** *	**18**	**0.012** *	
Thoracic	4		10		4		18		
Lumbar	4		11		7		22		
ASIA impairment scale^b^									
Complete A	3	0.181	10	0.772	3	0.121	18	0.581	
Incomplete B	5		11		4		22		
Incomplete C	4		11		5		19		
Incomplete D	5		10		5		21		

^a^ Mann-Whitney *U*-test.

^b^ Kruskal Wallis test.

* Significant at 95% confidence level.

The bold values are indicated as significant.

### Community integration questionnaire

The median score and *p* value for each domain score of community integration associated with each demographic characteristic are presented in Table 4. Participants with a paraplegic injury (thoracic skeletal level) and those with a complete A or incomplete B injury were significantly more independent (*p* = 0.013) in home integration activities such as grocery shopping, preparing meals, and childcare compared with other participants. Participants with a paraplegic injury were also significantly more independent (*p* = 0.040) in social integration activities such as paying bills, shopping, and visiting friends or relatives compared to those with a tetraplegic injury.

**Table 4:** Median scores and *P*-values of demographic and clinical factors for community integration questionnaire domains.

Socio-demographic factors	Home integration		Social integration	
	Median	*P*-value	Median	*P*-value
Age^a^				
<35 years	5	0.761	5	0.323
35 years & above	5		6	
Gender^a^				
Male	5	0.271	5	0.911
Female	6		5	
Education^b^				
Never attended school	5	0.422	5	0.512
Primary Education	6		5	
Secondary Education	5		5	
Higher Secondary	8		5	
Bachelor or above	5		5	
Type of injury^a^				
Paraplegic	6	**0.013** *	5	**0.040** *
Tetraplegic	5		4	
Skeletal level^b^				
Cervical	5	**0.010** *	4	0.551
Thoracic	6		5	
Lumber	5		5	
ASIA impairment scale^b^				
Complete A	6	**0.040** *	5	0.611
Incomplete B	6		6	
Incomplete C	4		4	
Incomplete D	5		5	

^a^ Mann-Whitney *U*-test.

^b^ Kruskal Wallis test.

* The bold values are indicated as significant at 95% confidence level.

### Multiple regression analysis

The multiple regression analysis of demographic and clinical variables on functional independence and community integration is presented in Table 5. Participants with a cervical [OR = 0.278, 95% CI: 0.114, 0.675] or thoracic level injury [OR = 0.398, 95% CI: 0.169, 0.935] were significantly less functionally independent compared with those with a lumbar level injury. People with a paraplegic injury were more likely to have a high level of functional independence [OR = 2.178, 95% CI: 1.380, 3.537] compared to those with a tetraplegic injury. Participants with an incomplete D SCI had higher functional independence scores [OR = 2.867, 95% CI: 1.333, 3.508] compared to those with a complete A SCI.

**Table 5:** Multiple regression analysis of demographic and clinical variables on functional independence and community integration.

Variables	Functional independence			Community integration					
	Adjusted OR	95% CI of OR		Home integration			Social integration		
				Adjusted OR	95% CI of OR		Adjusted OR	95% CI of OR	
		Lower	Upper		Lower	Upper		Lower	Upper
Age (ref: 35 years & above)									
<35 years	1.188	0.679	2.078	1.123	0.645	1.958	0.335	**0** **.** **041**	**0** **.** **883**
Occupation (ref: Service holder)									
Farmer	1.380	0.512	5.596	1.250	0.371	4.207	0.878	0.218	3.542
Day laborer	0.894	0.180	3.104	1.778	0.518	6.101	0.756	0.189	3.017
Housewife	1.171	0.612	6.540	1.583	0.481	5.316	0.950	0.258	3.505
Businessmen	2.230	0.309	3.802	1.449	0.484	5.210	1.270	0.317	5.097
Others	1.017	0.353	3.287	1.111	0.306	4.037	0.732	0.176	3.046
Monthly income (ref: >200 USD)									
<100 USD	1.875	0.483	6.266	1.389	0.419	4.600	0.563	0.137	2.302
100–200 p	0.761	0.591	6.954	1.169	0.385	3.549	0.737	0.195	2.790
Skeletal level (ref: Lumbar)									
Cervical	0.278	**0** **.** **114**	**0** **.** **675**	0.638	0.285	0.932	0.960	0.410	2.248
Thoracic	0.398	**0** **.** **169**	**0** **.** **935**	1.252	0.584	2.682	1.669	0.719	3.874
Type of injury (ref: Tetraplegic)									
Paraplegic	2.178	**1** **.** **380**	**3** **.** **537**	2.322	**1** **.** **260**	**3** **.** **896**	1.444	0.774	2.696
ASIA grade (ref: Complete A)									
Incomplete B	1.150	0.512	2.583	1.546	0.664	3.598	1.495	0.576	3.880
Incomplete C	1.037	0.326	3.302	2.719	**1** **.** **158**	**4** **.** **478**	2.705	0.745	9.826
Incomplete D	2.867	**1** **.** **333**	**3** **.** **508**	2.842	**1** **.** **297**	**4** **.** **169**	1.305	0.375	4.542

The bold values are indicated as significant.

Participants 35 year of age and above had significantly higher social integration compared to those below 35 years [OR 0.635: 95% CI: 0.141, 0.983]. People with a paraplegic injury were more likely to have good home integration [OR = 2.322, 95% CI: 1.260, 3.896] compared to those with a tetraplegic injury. Those with an incomplete Level C injury had significantly better home integration [OR = 2.719, 95% CI: 1.158, 4.478] compared those with a complete A SCI.

### Relationship between spinal cord injury measure (SCIM) and community integration questionnaire (CIQ)

The correlation analysis of the relationship between the SCIM and CIQ is presented in Table 6. These results indicate that higher levels of functional independence did not indicate better community integration. Although the relationship was not significant the result suggests a negative correlation between overall SCIM score and scores in both domains of CIQ.

**Table 6:** Correlation between spinal cord injury measure (SCIM) and community integration questionnaire (CIQ) (spearman correlation coefficient).

Variables	Community integration questionnaire (CIQ)			
	Home integration		Social integration	
	(*r*)	*p*	(*r*)	*p*
Spinal cord injury measure (SCIM)	−0.011	0.874	−0.094	0.185

## Discussion

The aim of this study was to determine if there was a relationship between the level of functional independence and community integration of people with SCI who had completed their rehabilitation at CRP. Functional independence and community integration were considered to be important measures as they can provide an indication of how effectively a person with SCI may participate in everyday activities when back in their community (37, 38).

It was expected that there would be a positive correlation between the results of the SCIM and CIQ; however, the findings suggest a potentially negative correlation between the overall SCIM scores and scores of both domains of CIQ. Loni et al. (7) found a positive relationship between improvements in functional independence because of inpatient rehabilitation and the life satisfaction, but no significant relationship between improvements in functional independence and the total quality of life score of people with SCIs. These researchers indicated that the short time frame of their study possibly impacted the results, and that measurements of quality of life and life satisfaction would benefit from being investigated over a longer period post-rehabilitation (7). In the current study it is likely that measuring community re-integration with the CIQ one-month post-discharge does not accurately capture how successful community re-integration has been. This is because it can take longer than a month for a person who has had a SCI to recalibrate and learn how to live with a SCI when they return to their community (7, 39).

It is also possible that the concepts measured on SCIM and the CIQ are not closely related in that a person's level of physical independence may not be a key factor in determining successful community reintegration. Based on the findings in their study Loni et al. (7) suggested that “non-physical factors, such as psychological, social and emotional factors, might play a pivotal role in shaping the quality of life and life satisfaction among individuals with spinal cord injury” (p. 6). Perhaps these same non-physical factors contribute to, and impact, the effectiveness of community integration.

Although there was no relationship found between SCIM and CIQ there were other findings that required exploration. The data presented in Tables 3–5 provide a summary of the relationship between demographic and clinical variables, and functional independence and home and social integration outcomes.

Age was found to be associated with social integration. Participants below 35 years of age had lower social integration compared to those who were aged 35 years and above. This is different to other research that indicates that community and social reintegration generally declines as people with a SCI age (5, 40). Charlifue and Gerhart (5) indicated that this is in line with typical aging, and, for this reason, it is not necessarily a cause for concern if people with a SCI “report that they are content with their levels of community integration” (p. 99). The finding that participants under the age of 35 years scored lower on social participation is different to the findings of other studies. Charlifue and Gerhart (5) stated that in many communities it is “individuals who are aging, regardless of whether or not they have a disability, [that] may be marginalized and not afforded opportunities to maximize community integration” (p. 92). In Bangladesh the lower social integration score may reflect that the participants under 35 years of age felt they were a burden to their family and to society. This may be due to the potential impact the SCI has on their health, family responsibilities and employment prospects. These participants may find it more challenging to transition “from the safety of the artificial hospital environment with the associated supports, both physical and social, and [plunge] into environments that are hostile towards people with disabilities” (11, p. 3845). Hence, these participants may be initially less content with their perceived levels of community integration.

It is not surprising to find that participants with a lower-level SCI (i.e., paraplegia) had significantly higher levels of functional independence and home and social integration compared with those who had a higher level of injury (i.e., tetraplegia). These findings can be explained by the relationship between the extent of muscle paralysis and sensory loss, and the higher risk of secondary complications, such as pressure ulcers, with the limitations on a person's functional independence and community integration (41, 42). Trgovevic and Milicevic found that people who had a paraplegic injury had better achievements in “home integration in comparison to achievements in persons with tetraplegia” (43, p. 189). However, in social integration these authors found no significant different. This is different to the findings in the present study. One reason for this difference may be the context in which participants were living, with participants in Bosnia and Herzegovina (43) experiencing better, environmental, health and social-welfare conditions compared with participants in Bangladesh. In their critical review of social and community participation following spinal cord injury, Barclay et al. (11) found a statistically significant relationship “between fewer barriers and increased community integration” (p. 16). It is possible that there may be more environmental barriers that a person with spinal cord injuries must negotiate when they return to live in a community in a developing country such as Bangladesh compared to returning to live in a community in a developed country such as Bosnia and Herzegovina. In relation to secondary complications, Lala et al. (44) indicated that people with pressure ulcers found it harder to take part in community activities than people without pressure ulcers. People with tetraplegia aremore at risk of pressure ulcers, which may partially explain why they had less mobility in their own homes and in the society compared to people with paraplegia (44).

The findings suggest that the more complete the spinal cord injury the lower the levels of functional independence and home and social integration. At the completion of inpatient rehabilitation at CRP, a participant with an AIS grade D (incomplete) SCI (refer to Table 2) had better functional outcomes, and higher home and social integration than a participant with an AIS grade A (complete) SCI (refer to Table 2). This finding is consistent with other research that found people who have an incomplete SCI often have better functional outcomes compared with those with a complete SCI (45). McKinley et al. (46) and Whiteneck et al. (47) stated that, completeness of damage and degree of neurological impairment are crucial indicators of functional prognosis and social reintegration following SCI.

There was an association found between participants' educational level and the domains of self-care and mobility in bed and action to prevent pressure sores according to the results of the SCIM scale. Participants who either never attended school or who had a bachelor degree had greater independence in this domain, compared with those who had attend primary, secondary and higher education. This finding is difficult to explain and requires further investigation. No other research was found that focused on the connection between education level and functional outcomes for people with SCI.

The findings of this study must be considered in the light of several limitations. Only a small number of women were included as participants in this study. Although this may reflect the proportion of men and women who were inpatients at CRP at the time of the study, the inclusion of more women in the study would lead to greater confidence in the findings being representative of the functional independence and home and social integration of women with SCI living in Bangladesh. The generalisability of the findings is also impacted by the fact that this study was conducted at only one institute in one country. A further limitation is that the SCIM was completed at the end of rehabilitation and the completion of the CIQ was at one-month post-discharge. Hence, the findings only provide a baseline for functional outcome and community integration measures at around the time of discharge.

Future research could focus on following up participants at regular intervals post-discharge to monitor their home and social integration to identify any factors that may impact on a person's community integration over time. There is some evidence to suggest that a person's functional independence, as determined by the SCIM, is relatively stable over time (48). However, although functional independence may be stable, a person's community integration could change over time, particularly because of secondary complications and ageing (5, 21, 23). As functional independence may not be a suitable indicator of community integration it may be more relevant to monitor community integration along with other factors, such as perceptions of quality of life as was done my Moller et al. (48) and proposed by Rahman et al. (20), to identify support and intervention needs of people with SCI post-discharge. There would be benefit in increasing the proportion of women who participate in the study to determine if there are functional independence and/or social and home integration outcomes that are more specific to women.

## Conclusion

Although no statistically significant relationship was found between functional independence and community integration one month post-discharge, the findings from this study provide a baseline for measuring and monitoring the level of functional independence and community integration of people with SCI who completed their rehabilitation at CRP. The level of functional independence was related to the level and completeness of spinal cord injury, and age and level of education were factors identified as having a significant relationship with level of home and social community integration. It has been suggested that functional independence may not change over time; however, the level of community integration may change due to multiple factors including age, secondary complications, social support, and quality of life. This could mean that a measure of functional independence is not a reliable indicator of community integration. To objectively monitor a person's community integration over time post-discharge it may be more relevant to use a tool such as the CIQ, with a measure of their QOL and/or life satisfaction. This would at least provide a measure of a person's contentment with their life that can be considered alongside their community integration outcomes. This may contribute to identifying any support and interventions a person may require that will contribute to successful community integration.

## Data Availability

The raw data supporting the conclusions of this article will be made available by the authors, without undue reservation.
